# Oxidative stress gene expression, DNA methylation, and gut microbiota interaction trigger Crohn’s disease: a multi-omics Mendelian randomization study

**DOI:** 10.1186/s12916-023-02878-8

**Published:** 2023-05-11

**Authors:** Shu Xu, Xiaozhi Li, Shenghong Zhang, Cancan Qi, Zhenhua Zhang, Ruiqi Ma, Liyuan Xiang, Lianmin Chen, Yijun Zhu, Ce Tang, Arno R. Bourgonje, Miaoxin Li, Yao He, Zhirong Zeng, Shixian Hu, Rui Feng, Minhu Chen

**Affiliations:** 1 Department of Gastroenterology, The First Affiliated Hospital, Sun Yat-Sen University, Guangzhou, Guangdong China; 2 Microbiome Medicine Center, Division of Laboratory Medicine, Zhujiang Hospital, Southern Medical University, Guangzhou, Guangdong China; 3 Department of Computational Biology for Individualised Medicine, Centre for Individualised Infection Medicine & TWINCORE, Joint Ventures Between the Helmholtz Centre for Infection Research and the Hannover Medical School, Hannover, Germany; 4 Changzhou Medical Center, The Affiliated Changzhou No. 2 People’s Hospital of Nanjing Medical University, Nanjing Medical University, Changzhou, Jiangsu China; 5 Department of Cardiology, The First Affiliated Hospital of Nanjing Medical University, Nanjing Medical University, Nanjing, Jiangsu China; 6 Institute of Precision Medicine, The First Affiliated Hospital, Sun Yat-Sen University, Guangzhou, Guangdong China; 7 Department of Gastroenterology and Hepatology, University of Groningen, University Medical Center Groningen, Groningen, The Netherlands; 8 Zhongshan School of Medicine, Center for Precision Medicine, Sun Yat-Sen University, Guangzhou, Guangdong China; 9 Department of Gastroenterology, Guangxi Hospital Division of The First Affiliated Hospital, Sun Yat-Sen University, Nanning, Guangxi China

**Keywords:** Oxidative stress, Crohn’s disease, Integrative omics, Mendelian randomization

## Abstract

**Background:**

Oxidative stress (OS) is a key pathophysiological mechanism in Crohn’s disease (CD). OS-related genes can be affected by environmental factors, intestinal inflammation, gut microbiota, and epigenetic changes. However, the role of OS as a potential CD etiological factor or triggering factor is unknown, as differentially expressed OS genes in CD can be either a cause or a subsequent change of intestinal inflammation. Herein, we used a multi-omics summary data-based Mendelian randomization (SMR) approach to identify putative causal effects and underlying mechanisms of OS genes in CD.

**Methods:**

OS-related genes were extracted from the GeneCards database. Intestinal transcriptome datasets were collected from the Gene Expression Omnibus (GEO) database and meta-analyzed to identify differentially expressed genes (DEGs) related to OS in CD. Integration analyses of the largest CD genome-wide association study (GWAS) summaries with expression quantitative trait loci (eQTLs) and DNA methylation QTLs (mQTLs) from the blood were performed using SMR methods to prioritize putative blood OS genes and their regulatory elements associated with CD risk. Up-to-date intestinal eQTLs and fecal microbial QTLs (mbQTLs) were integrated to uncover potential interactions between host OS gene expression and gut microbiota through SMR and colocalization analysis. Two additional Mendelian randomization (MR) methods were used as sensitivity analyses. Putative results were validated in an independent multi-omics cohort from the First Affiliated Hospital of Sun Yat-sen University (FAH-SYS).

**Results:**

A meta-analysis from six datasets identified 438 OS-related DEGs enriched in intestinal enterocytes in CD from 817 OS-related genes. Five genes from blood tissue were prioritized as candidate CD-causal genes using three-step SMR methods: *BAD*, *SHC1*, *STAT3*, *MUC1*, and *GPX3*. Furthermore, SMR analysis also identified five putative intestinal genes, three of which were involved in gene–microbiota interactions through colocalization analysis: *MUC1*, *CD40*, and *PRKAB1*. Validation results showed that 88.79% of DEGs were replicated in the FAH-SYS cohort. Associations between pairs of *MUC1*–*Bacillus aciditolerans* and *PRKAB1*–*Escherichia coli* in the FAH-SYS cohort were consistent with eQTL–mbQTL colocalization.

**Conclusions:**

This multi-omics integration study highlighted that OS genes causal to CD are regulated by DNA methylation and host-microbiota interactions. This provides evidence for future targeted functional research aimed at developing suitable therapeutic interventions and disease prevention.

**Supplementary Information:**

The online version contains supplementary material available at 10.1186/s12916-023-02878-8.

## Background

Crohn’s disease (CD) is a type of chronic and relapsing inflammatory bowel disease (IBD) that affects the gastrointestinal tract and is accompanied by extraintestinal manifestations and perianal diseases [1]. Although the etiology of CD remains unclear, a complex interplay between genetic variation, environmental factors, immune dysfunction, and intestinal microbiota is believed to underlie disease pathogenesis [2]. Unraveling the complexity behind this interplay may provide crucial insights into CD pathogenesis and expose potential targets for therapeutic interventions and disease prevention.

Oxidative stress (OS) is defined as an imbalance between oxidants and antioxidants in favor of the oxidants leading to a disruption of redox signaling and control and/or molecular damage [3]. Multiple OS-related genes contribute to the complex multifactorial pathophysiology in CD [4, 5]. For example, genetic polymorphisms of the inducible nitric oxide synthase gene (encoded by *NOS2A*) are associated with IBD susceptibility accompanied by increased gene expression, suggesting a vital role of genetic effects on OS genes in CD [6]. The nicotinamide adenine dinucleotide phosphate oxidase genes *NOX1* and *DUOX2* also play a key role in mediating reactive oxygen species (ROS) generation. Overexpression of these genes is involved in impaired intestinal barrier integrity, microbial dysbiosis, and bacterial invasion, highlighting the association between host OS signaling and gut microbiota in CD [7–10]. Moreover, DNA methylation (DNAm) modulates redox homeostasis by regulating gene expressions of *NRF2*, *HIF1A*, and related proteins [11–14]. However, few studies have addressed whether OS has a causative role in triggering CD or merely inflicts collateral tissue damage alongside intestinal inflammation. Studying the underlying disease mechanisms of OS-related genes may help identify potential pathogenetic factors and redox-related therapeutic targets for IBD [15].

Although a growing number of studies have suggested relevant OS genes in CD, no study has comprehensively and systematically identified their potential causal association with this disease. Genome-wide association studies (GWASs) have been employed to identify genomic loci containing OS genes associated with CD [16, 17]. However, the top associated variants may not be causal because of the complicated linkage disequilibrium (LD) structure of genomes [18, 19]. Moreover, these genetic variants can potentially regulate DNAm, gene expression, protein levels, and the abundance of gut microbiota [20, 21]. Integration of multi-omics is an emerging approach in the post-GWAS era to identify critical regulators for exploring therapeutic targets in CD [22]. For example, summary data-based Mendelian randomization (SMR) that integrates IBD GWAS data with expression quantitative trait loci (eQTLs) has been developed to prioritize causal variants mediated by gene expression in the blood [20]. However, the causal OS genes in CD-affected tissues and their interactions with gut microbiota are poorly understood [23–25].

This study presents a multi-omics-based Mendelian randomization (MR) study to identify the putative causal effects and molecular mechanisms of OS genes in CD using blood and intestinal tissues. A sizable intestinal transcriptome meta-analysis was performed to identify differentially expressed CD-related OS genes. Utilizing SMR methods, we integrated the largest CD GWAS summary statistics with eQTLs and DNA methylation QTLs (mQTLs) in the blood. Furthermore, up-to-date intestinal eQTLs and fecal microbial QTLs (mbQTLs) were first integrated into the current analysis to uncover the potential interactions between host OS genes and gut microbiota. Two additional MR methods were used as sensitivity analyses to test the heterogeneity. Finally, the putative results were then partially replicated in an independent multi-omics cohort.

## Methods

### Study design and data resources

**Fig. 1:**
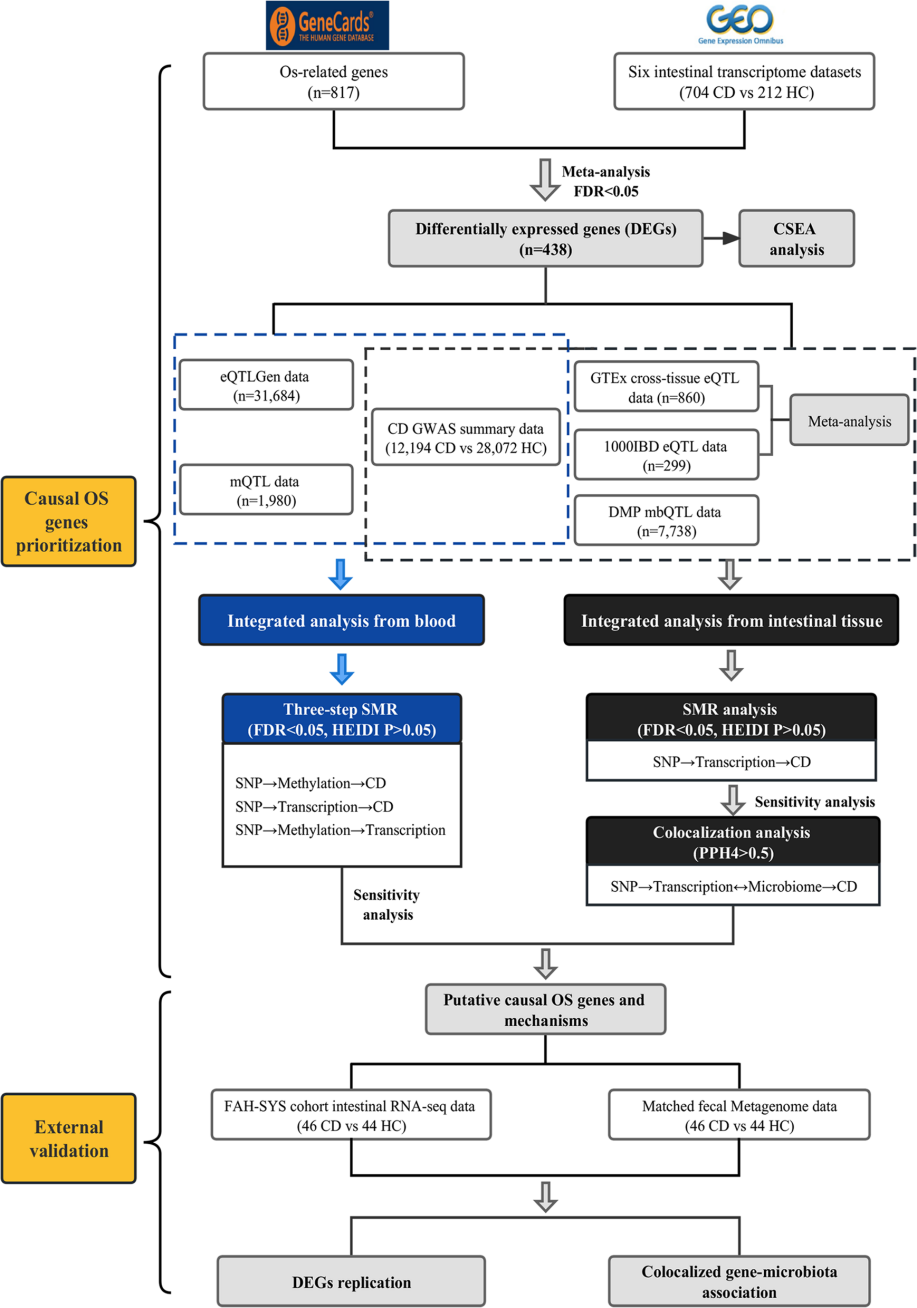
Workflow of the study. A series of analyses was conducted to identify candidate causal oxidative stress (OS) genes associated with Crohn’s disease (CD) onset. OS-related genes were extracted from the GeneCards database. Six intestinal transcriptome datasets including patients with CD and healthy controls (HCs) were obtained from the GEO database and meta-analyzed to identify differentially expressed CD-related OS genes, followed by cell type-specific expression analysis (CSEA). Integration of GWAS summaries and *cis*-eQTLs/*cis*-mQTLs data from the blood by using three-step SMR methods prioritized putative blood OS genes and their regulatory elements associated with the risk of CD (SMR FDR < 0.05; HEIDI test *P* > 0.05). Sensitivity analyses were performed after the primary SMR to test the heterogeneity (Cochran *Q* statistic implemented in MR-Egger and inverse variance weighting (IVW) method,* P* > 0.05 indicates no heterogeneity exists). Moreover, we meta-analyzed the intestinal *cis*-eQTLs from two public summaries (GTEx and 1000IBD) and further integrated the meta-intestinal *cis*-eQTLs with fecal mbQTLs from the Dutch Microbiome Project (DMP) to uncover the potential interactions between OS genes and gut microbiota through SMR, sensitivity, and colocalization analysis (SMR FDR < 0.05; HEIDI test *P* > 0.05; Cochran *Q P* > 0.05; colocalization PPH4 > 0.5). An external First Affiliated Hospital of Sun Yat-sen University (FAH-SYS) inflammatory bowel disease (IBD) multi-omics cohort with paired intestinal bulk RNA-seq and fecal metagenomics data was used to validate the differentially expressed genes (DEGs). The directional associations between colocalized gene-microbiota were investigated as complementary evidence of OS gene-microbiota interactions

Figure 1 describes the design of this study. OS-related genes were extracted from the GeneCards database (v5.10, https://www.genecards.org) using the keyword “oxidative stress” with a relevance score ≥ 7 according to previous methods [26–28]. Six publicly available transcriptome datasets containing intestinal biopsies from patients with CD and healthy controls (HCs) were obtained from the Gene Expression Omnibus (GEO) database [29–39] and meta-analyzed to identify differentially expressed genes (DEGs) related to OS in CD. GWAS summary statistics for CD were derived from a meta-analysis of two separate IBD GWASs, yielding a sample size of 12,194 patients with CD and 28,072 HCs based on European populations [40]. Blood eQTL summary statistics of OS genes were obtained from eQTLGen including the genetic data of blood gene expression in 31,684 individuals derived from 37 datasets [41]. Blood mQTL summary data were generated from a meta-analysis of two European cohorts: the Brisbane Systems Genetics Study (*n* = 614) and the Lothian Birth Cohorts (*n* = 1366) [19]. Intestinal eQTL data were from the Genotype-Tissue Expression (GTEx) project (*n* = 860) [42] and the 1000IBD cohorts (*n* = 299) [43]. The current study focused only on *cis*-eQTLs and *cis*-mQTLs, which constituted single nucleotide polymorphisms (SNPs) within a 1-Mb distance from the start and end of the gene. Fecal mbQTL data was generated from the Dutch Microbiome Project (DMP) study, which included data from 7738 individuals to assess the host genetic effects on the gut microbiota [44].

For external validation, 46 treatment-naïve patients with CD and 44 HC subjects were prospectively recruited from the First Affiliated Hospital of Sun Yat-sen University (FAH-SYS) IBD multi-omics cohort. Paired intestinal biopsies and stool specimens were collected for RNA sequencing (RNA-seq) and shotgun metagenomic sequencing, respectively.

More detailed information on study datasets for this study is provided in Additional file 1: Supplementary Methods [19, 29–45].

### Statistical analysis

#### Meta-analysis of DEGs

Intestinal DEGs related to OS in patients with CD and HCs were analyzed using a linear regression model while adjusting for age, sex, body mass index, and medication usage if metadata were available. To increase statistical power, we pooled ileal and colonic biopsies and added tissue location as a covariate in linear models. DEGs were analyzed separately in six gene expression datasets, followed by a fixed-effects meta-analysis using the R package *metafor*.

#### Meta-analysis of intestinal *cis*-eQTLs

To include as many intestinal *cis*-eQTLs as possible, we first performed a meta-analysis using the meta-analysis of *cis*-eQTL in the correlated sample (MeCS) method across the transverse colon, sigmoid colon, and small intestine of the GTEx results for OS genes, considering the sample overlaps. The intestinal *cis*-eQTLs from the 1000IBD cohorts were highly comparable to those detected in the non-disease GTEx dataset (consistency rates > 97%) [43]. Therefore, we used the conventional inverse-variance-weighted meta-analysis in SMR (v1.3.1) for the two independent datasets.

#### SMR and colocalization analysis

SMR multi-tools have been established to detect whether the effect of SNPs on the phenotype is mediated by molecular traits such as gene expression, DNAm, and gut microbiota. Colocalization analysis aimed to investigate the overlapping variants likely responsible for different traits. The integration of data from GWAS with other molecular QTL data by SMR or colocalization improved the detection of candidate causal SNPs via specific pathways.

Blood tissue analysis used the SMR multi-tool to determine the causal inference of OS genes and the 1000 Genomes European reference to calculate LD. A three-step SMR analysis was performed: (1) SNPs were instruments, blood gene expressions were exposure, and CD was the outcome; (2) SNPs were instruments, blood DNAms were exposure, and CD was the outcome; (3) SNPs were instruments, blood DNAms were exposure, and blood gene expressions were the outcome. The third step included only significant signals from steps 1 and 2. The final candidate signals were defined as those that (1) passed all three-step SMR false discovery rate (FDR) < 0.05; (2) were suggestively significant genome-wide (*P* < 1 × 10^−5^) in all eQTLs, mQTLs, and GWAS; and (3) exhibited heterogeneity in the dependent instrument (HEIDI) test results with *P* > 0.05.

Intestinal tissue analysis used the SMR multi-tool to determine the causal inference between GWAS and *cis*-eQTLs. This included SNPs as instruments, intestinal gene expressions as exposure, and CD as the outcome (SMR FDR < 0.05, HEIDI *P* > 0.05, *cis*-eQTLs, and GWAS *P* < 1 × 10^−5^).

Sensitivity analyses were conducted after completing the primary SMR analyses with two additional MR methods. We tested for heterogeneity across the individual causal effects using Cochran *Q* statistic implemented in both MR–Egger and inverse variance weighting (IVW) methods. The *P* value of Cochran’s *Q* test < 0.05 or the HEIDI < 0.05 indicates the existence of heterogeneity.

Colocalization was chosen to assess the potential interactions between intestinal gene expression and microbiota because of the limited power to assess the causality between gut microbiota and diseases [46, 47]. It is a method to assess the presence of a shared causal variant in the region for two traits. Analysis was performed using the *coloc* R package with PPH4 > 0.5 as the threshold for the shared genetic effects between the two traits [48, 49].

#### Replication in the FAH-SYS IBD multi-omics cohort

(1) Significant DEG results from the six-dataset meta-analysis were selected to test between patients with CD and healthy controls in our cohort, and (2) the significant gene expression–microbial pathway pairs from the colocalization analysis were selected. The correlations between intestinal gene expression and pathway-related microbial taxa (genus- and species-level) abundance were then assessed using a linear regression model adjusted for age, sex, and body mass index. Pathway-related microbial taxa were defined using MetaCyc (v24.0) *pathways_to_organisms* definitions (https://metacyc.org/) containing the potential microbiota involved in each pathway. Only taxa with a present rate > 10% in the FAH-SYS IBD cohort were kept for the analysis resulting in three species and two genera. More specifically, CRNFORCAT.PWY was mainly predicted from *Methylobacterium* and *Cupriavidus*, P164.PWY and PYRIDNUCSYN.PWY were mainly predicted from *Escherichia coli*, PWY.5101 was mainly predicted from *E. coli* and *Saccharomyces cerevisiae*, and PWY.7237 was mainly predicted from *E. coli* and *Bacillus aciditolerans*, while no PWY0.845-associated taxa survived after filtering. A *P* value < 0.05 was considered as the significant threshold considering the small sample size of the FAH-SYS IBD cohort.

Unless otherwise stated, statistical significance was defined as FDR < 0.05 by the Benjamini–Hochberg (BH) method.

### Cell type-specific enrichment and regulatory component annotation

The Cell type-Specific Enrichment Analysis DataBase (CSEA-DB, https://bioinfo.uth.edu/CSEADB/) was used to investigate whether intestinal DEGs were specific to any cell type. Among 126 general cell types from 111 tissues, we focused on cell types present in the intestine, which resulted in seven tissues and 23 general cell types, after multiple testing corrections (BH method, FDR < 0.05).

The regulatory signature enrichment of DNAm sites was assessed using eFORGE (http://eforge.cs.ucl.ac.uk/), including chromatin status (active and inactive) and histone marker (H3K4me1 and H3K4me3) annotation. The regions of the individual DNAm sites were annotated at http://grch37.ensembl.org/.

## Results

### Meta-analysis of differentially expressed OS genes between patients with CD and HCs

**Fig. 2:**
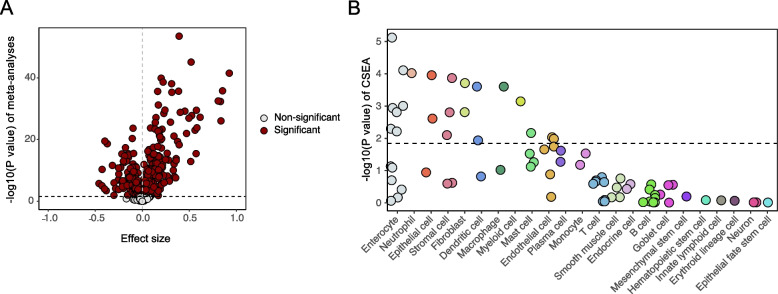
Meta-analysis of six intestinal gene expression datasets between patients with CD and HCs. **A** In total, 708 out of 817 genes presented in all six intestinal transcriptome data were assessed for expression differences between patients with CD and HCs. The volcano plot shows the meta effect sizes on the *x*-axis while the *y*-axis indicates the − log10-transformed meta *P* values. Red dots are the 438 significant differentially expressed genes (DEGs), and gray dots represent non-significantly expressed genes. The dashed line indicates the significant threshold with FDR < 0.05 corrected for the number of gene tests. **B** Cell type-Specific Enrichment Analysis DataBase was used to investigate whether the intestinal DEGs were specific to any cell type in the small intestine and colon. The *x*-axis indicates the cell types derived from the intestinal tissue and blood. Dots represent 77 small intestine and colon cell types annotated by 23 general classifications descending by order of significance. The dashed line is the significant threshold with FDR < 0.05

To understand the role of OS genes in CD, a total of six gene expression datasets (three microarray datasets and three RNA-seq datasets) were included to compare RNA expression in the intestinal tissues of patients with CD (*n* = 704) and HCs (*n* = 212) through a meta-analysis (Additional file 2: Table S1). In total, 817 OS-related genes with a relevance score ≥ 7 were downloaded from GeneCards (the “Methods” section; Additional file 2: Table S2). Subsequently, 438 OS genes were differentially expressed between CD and control tissues (FDR < 0.05) according to the meta-analysis (Fig. 2A and Additional file 2: Table S3). The top five genes prioritized by effect size were *DUOX2*, *MMP3*, *S100A8*, *MMP1*, and *IL1B*, which are all reported to be associated with intestinal inflammation in IBD [7, 50–52]. Furthermore, we conducted cell type-specific expression analysis (CSEA) of these DEGs. OS-related DEGs were significantly enriched in intestinal enterocytes (FDR = 0.002) among the 23 general cell classifications (Fig. 2B; the "Methods" section; Additional file 2: Table S4), suggesting an essential role of the epithelial cells in regulating intestinal OS and maintaining mucosal homeostasis.

### Integration of GWAS and OS-related eQTL/mQTL data from the blood

Given such an amount of OS DEGs (53.61%, 438 out of 817) observed in patients with CD compared with HCs, we then hypothesized that gene expression may explain the plausible causality in the disease. Additionally, DNAm located in promoters or enhancers commonly influences the regulation of disease-associated target genes [19]. Therefore, we aimed to identify candidate causal genes for CD and explore their possible underlying epigenetic mechanism of gene regulation in the blood. A three-step SMR method was used, and only the significant results in all three SMR analyses that passed sensitivity checks were interpreted as suggestive causal genes (the “Methods” section). Herein, 438 OS-related DEG *cis*-eQTLs (94,037 SNP–gene pairs) and their *cis*-mQTLs (52,761 SNP–CpG sites) were integrated with the largest available GWAS summary statistics for CD.

In concrete, the integration of eQTL results from the eQTLGen Consortium (*n* = 31,684) and CD GWAS summary statistics resulted in 16 OS-related genes (FDR < 0.05, HEIDI *P* > 0.05, and Cochran’s *Q P* > 0.05) (Additional file 2: Table S5). Meanwhile, we identified 665 DNAm probes (near 127 genes within 1 Mb) by integrating the same CD GWAS results and mQTL summary statistics from the meta-analysis of Brisbane Systems Genetics Study and Lothian Birth Cohorts (*n* = 1980) (Additional file 2: Table S6). Further integration analysis of putative CD-causal *cis*-eQTL and *cis*-mQTL data prioritized eight DNAm probes potentially regulating five neighboring genes: *BAD*, *SHC1*, *STAT3*, *MUC1*, and *GPX3* (SMR FDR < 0.05, HEIDI *P* > 0.05, and Cochran’s *Q P* > 0.05) (Additional file 2: Table S7). As expected, these CpG sites were significantly enriched in the transcription start sites (TSSs) of peripheral blood cells, including primary hematopoietic stem cells (FDR = 1.65 × 10^−16^), primary T helper memory blood cells (FDR = 1.94 × 10^−15^), and primary mononuclear blood cells (FDR = 4.79 × 10^−12^) (top three significance) (Additional file 3: Fig. S1; Additional file 2: Table S8).

### Putative CD-causal genes mediated by blood methylation regulation on gene expression

**Fig. 3:**
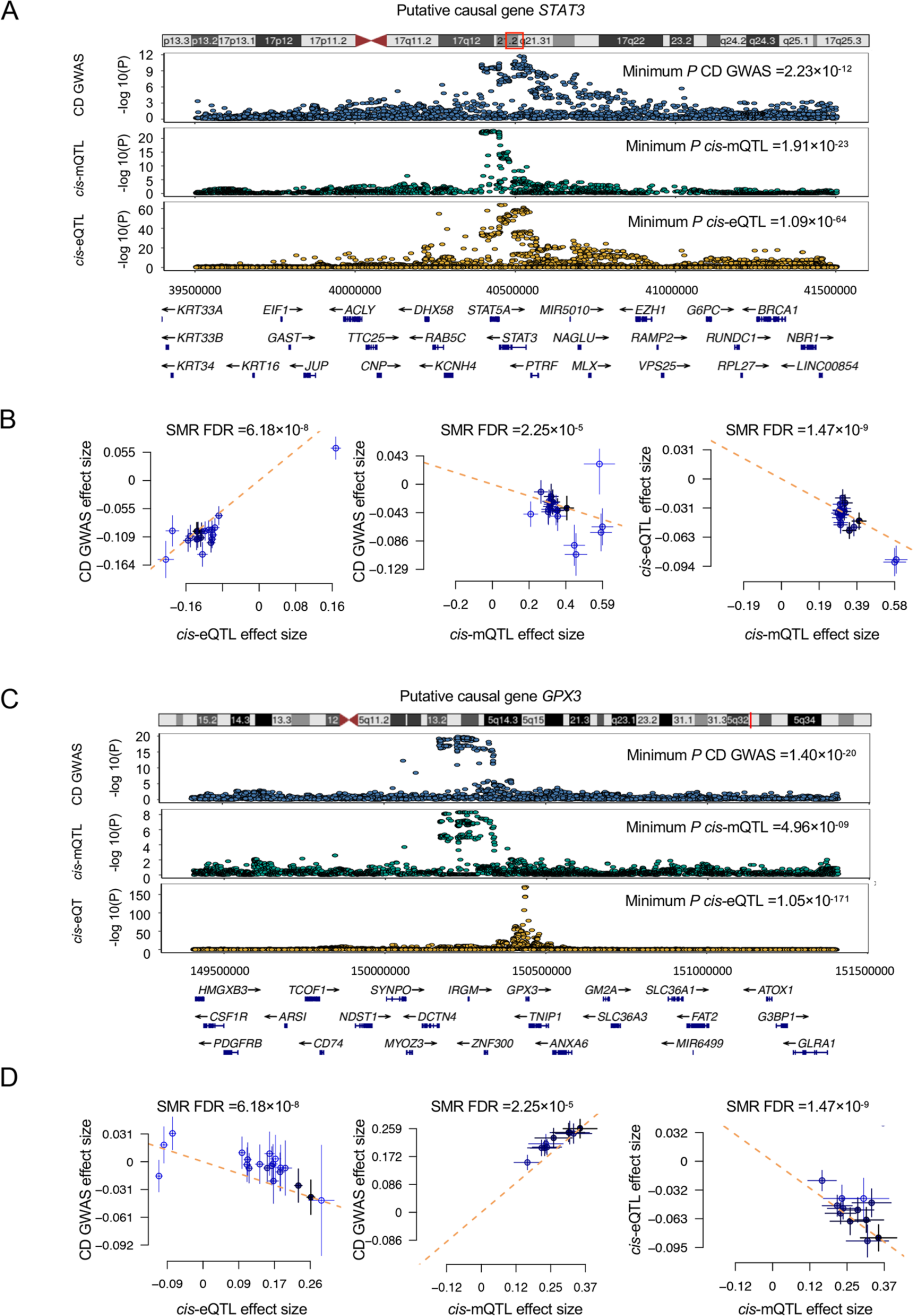
Three-step SMR analysis prioritized putative causal OS genes and mechanisms in CD using blood tissue. Examples of well-known and novel CD-causal OS genes. **A**, **C** Locus zoom plots showing the consistent genetic effects from CD GWAS, *cis*-mQTL, and *cis*-eQTL nearby *STAT3* and *GPX3* (from upper to lower panels, all minimum *P* < 1 × 10^−5^). **B**, **D** Three-step SMR indicating significant causal relationships between gene expressions and CD onset mediated by methylation (all three-step SMR FDR < 0.05, HEIDI test *P* > 0.05). From left to right: SMR between gene expression and CD GWAS, SMR between gene methylation and CD GWAS, and SMR between gene methylation and expression

Our three-step SMR analysis prioritized *STAT3*, a well-known redox-regulated gene, and its expression is strictly affected by the intracellular redox environment [53]. This study showed that the SNP signals associated with *STAT3* were significant across the data from CD GWAS, eQTL, and mQTL studies. The DNAm probe cg06422947 was found to be located in the enhancer region, 427 kbp upstream of *STAT3*. The methylation level of this site showed a negative effect on *STAT3* expression (beta_SMR_ =  − 0.11) and CD onset (beta_SMR_ =  − 0.09), while the *STAT3* expression level was positively associated with the disease (beta_SMR_ = 0.70). Together, our results suggest a putative mechanism wherein a lower DNAm level at the enhancer region of *STAT3* upregulates the expression of *STAT3* and subsequently increases CD risk (Fig. 3A, B).

Another key example is *GPX3* (Fig. 3C, D) which belongs to the glutathione peroxidase family and catalyzes the reduction of organic hydroperoxides and hydrogen peroxide by glutathione, thereby protecting cells against oxidative damage [54]. We found that DNAm probe cg08580836, located in the promoter region, was causally negatively associated with *GPX3* expression (beta_SMR_ =  − 0.24). Consistently, higher *GPX3* gene expression (beta_SMR_ =  − 0.15) and lower methylation levels (beta_SMR_ = 0.74) potentially decreased the risk of CD. Thus, the putative mechanism could be that the genetic variants upregulate *GPX3* expression by influencing the promoter DNAm status, showing a protective effect on CD onset.

### Integration of GWAS and OS-eQTL/mbQTL data from intestinal tissue

Genetic effects on gene expression vary across blood and intestine tissues, which could reflect different CD-causal genes [55]. Moreover, host genetics and gut microbiota are known to play critical roles in CD [56]. Intestinal tissues are in direct contact with gut microbes and sense local changes in OS levels; therefore, we hypothesized that integrating *cis*-eQTLs and mbQTLs from intestinal tissue would provide novel candidate targets with putative host–microbiota interactions. First, we performed a meta-analysis of *cis*-eQTL data from three intestinal tissues (sigmoid colon, transverse colon, and small intestine) obtained from the GTEx project (*n* = 860) adjusting for sample overlap. This was followed by a meta-analysis with intestinal *cis*-eQTL data from the 1000IBD project (*n* = 299). The final meta-analysis identified 43,975 *cis* SNP–gene pairs corresponding to 392 OS-related genes (FDR < 0.05) (Additional file 2: Table S9). The SMR analysis demonstrated the potential causal role of five intestinal-expressed genes in CD (SMR FDR < 0.05, HEIDI* P* > 0.05, and Cochran’s *Q P* > 0.05): *MUC1*, *CD40*, *PARK7*, *PRKAB1*, and *NDUFS1* (Additional file 2: Table S10).

To further explore the role of intestinal OS genes from the perspective of host–microbiota interactions, we integrated mbQTL summary statistics with putative CD-causal *cis-*eQTLs by colocalization analysis. This analysis was assumed to determine the probability that the genetic determinants of mucosal gene expression were shared with gut microbiota. SMR was not used because of power issues suffering from the moderate effects of host genetics on the gut microbiota [46, 47]. In total, six gene expression–microbial pathway pairs were detected at the threshold of PPH4 > 0.5, including three of the above genes, *MUC1*, *CD40*, and *PRKAB1* (Additional file 2: Table S11; Additional file 4: Fig. S2).

### Putative CD-causal genes involved in intestinal gene–microbiota interactions

**Fig. 4:**
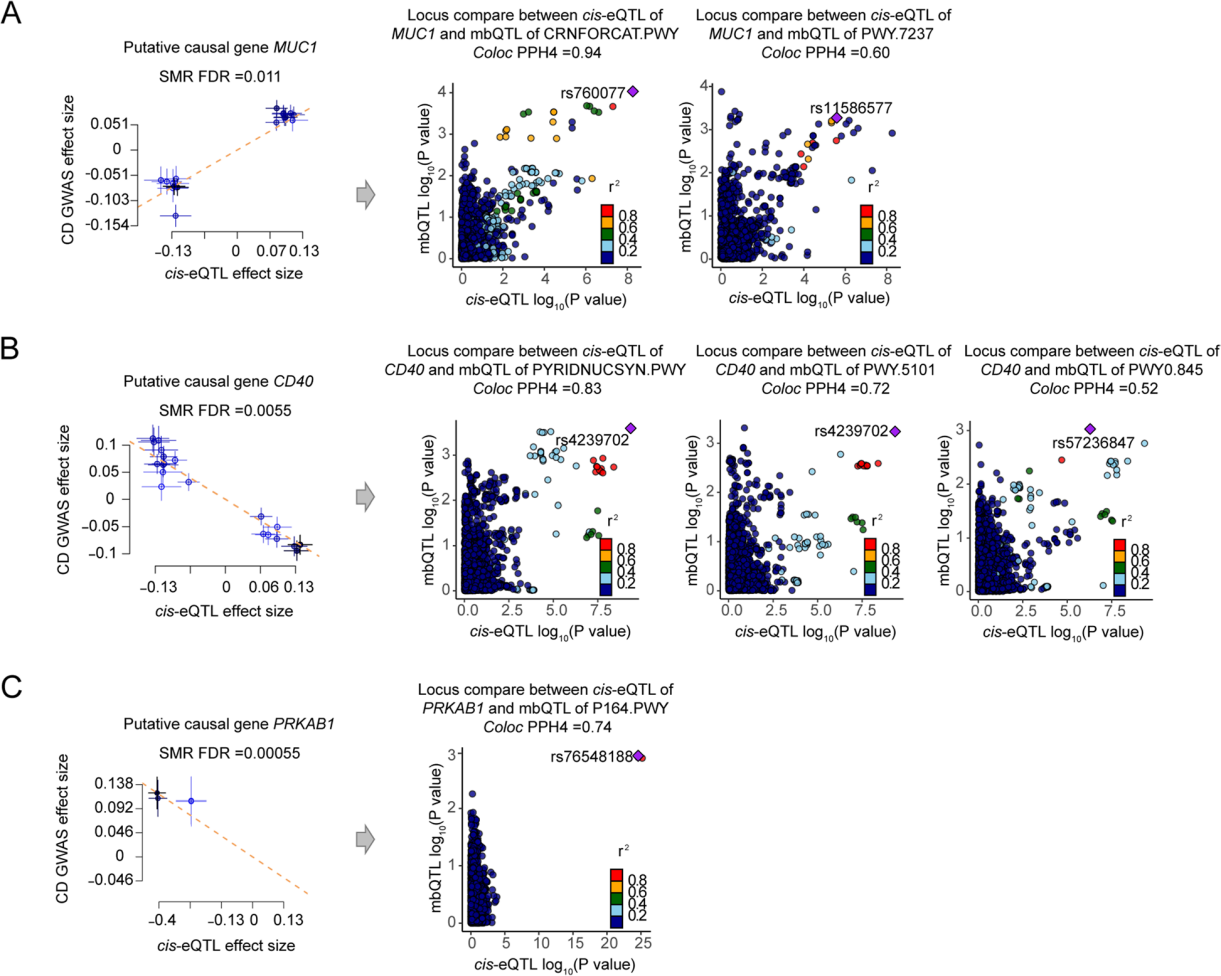
SMR and colocalization analyses prioritized intestinal causal OS genes and interactions with gut microbial pathways in CD. The left panels indicate the SMR between gene expressions and CD GWAS (all SMR FDR < 0.05; HEIDI test *P* > 0.05), while the right panels show the locus comparisons between *cis*-eQTLs and mbQTLs by colocalization analysis (all PPH4 > 0.5). The *r* ^2^ value indicates the linkage disequilibrium (LD) between the variants and the top SNPs. **A**–**C** The genes of *MUC1*, *CD40*, and *PRKAB1*, respectively

We prioritized *MUC1* as a candidate OS causal gene in CD intestinal tissues associated with the gut microbiota based on SMR and colocalization analysis (Fig. 4A). Our study showed that elevated *MUC1* expression likely played a causal role in CD onset (beta_SMR_ = 0.57). Furthermore, SNPs regulating *MUC1* expression might also affect the microbial metabolic functions given the colocalization analysis. Concretely, the creatinine degradation (CRNFORCAT.PWY; PPH4 = 0.94) and myo-inositol degradation (PWY.7237; PPH4 = 0.60) microbiota metabolic pathways, which are associated with decreased inflammation or short-chain fatty acids production, shared genetic effects with *MUC1* expression. Our findings suggest that genetic variation in *MUC1* might simultaneously regulate its gene expression and the production of microbiota-derived metabolites, thus increasing the risk of CD.

The *CD40* gene for CD is another example involved in intestinal gene–microbiota interactions (Fig. 4B). *CD40* and its ligand (*CD40L*) are associated with ROS production in immune and endothelial cells [57, 58]. They constitute the second activation signal of macrophages and enhance its activation and the resultant production of potential antimicrobial peptides as well as ROS, nitric oxide, and related metabolites [59]. This study showed a protective effect of CD40 expression against CD (beta_SMR_ =  − 0.62), which was also found in the case of other immune-related disease studies [60]. Three microbial pathways were associated with *CD40* gene expression under genetic regulation: nicotinamide adenine dinucleotide biosynthesis from aspartate (PYRIDNUCSYN.PWY; PPH4 = 0.83), L-isoleucine biosynthesis (PWY.5101; PPH4 = 0.72), and the superpathway of pyridoxal 5′-phosphate biosynthesis and salvage (PWY0.845; PPH4 = 0.52). These microbiota-derived substances have also been reported to be correlated with intestinal inflammation [61–65]. For instance, nicotinamide adenine dinucleotide can be produced by certain gut bacteria from L-aspartate [66], which is associated with gut inflammation in IBD [63, 64]. L-isoleucine supplementation alleviates intestinal inflammation in vivo and in vitro [65]. Thus, we hypothesize a mechanism by which genetic variants regulate *CD40* expression and interact with inflammation-related microbial activities and therefore contribute to CD pathogenesis.

Another candidate causal gene is *PRKAB1* (Fig. 4C). This study showed that *PRKAB1* expression was also negatively associated with the risk of developing CD (beta_SMR_ =  − 0.30). Additionally, its expression shared genetic effects with microbial purine nucleobase degradation (P164.PWY; PPH4 = 0.74), indicating a potential interaction between gene expression and intestinal microbial nucleotide metabolism.

### External replication of OS DEGs and plausible gene–microbiota interactions in the FAH-SYS cohort

**Fig. 5:**
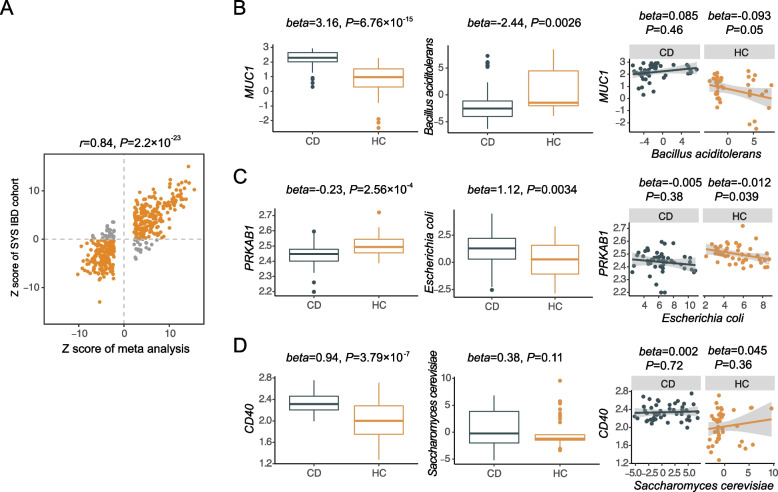
External cohort validation. First Affiliated Hospital of Sun Yat-sen University (FAH-SYS) cohort with both intestinal bulk RNA-seq and fecal metagenomics data included in validation analysis. **A** A total of 388 out of 437 (88.79%) differentially expressed genes (DEGs) identified from the meta-analysis show consistent changes between patients with CD and HCs (Spearman rank’s correlation coefficient *r*_*s*_ = 0.84, *P* = 2.2 × 10^−23^). The *x*- and *y*-axes indicate the *Z* score estimated from the meta-analysis and FAH-SYS cohort, respectively. Yellow dots represent the 388 consistent DEGs, while gray dots are non-validated. **B**–**D** Validation of DEGs, pathway-related taxa, and the association between taxa and gene expressions, respectively (from left to right). The upper to lower panels are for the *MUC1*, *PRKAB1*, and *CD40* genes, respectively. The taxa probably involved in PWY.7237, P164.PWY, and PWY.5101 were selected from MetaCyc database annotation

To confirm the prioritized genes above, we first validated the DEG results from the meta-analysis using intestinal transcriptomic sequencing data in an independent FAH-SYS cohort (CD *n* = 46; HC *n* = 44; the “Methods” section; Additional file 2: Table S12). A lenient significance threshold (*P* < 0.05) was adopted considering the limited sample size. In total, 437 out of 438 intestinal DEGs from the meta-analysis were tested between patients with CD and HCs. Of note, 388 (88.79%) genes were consistently upregulated or downregulated under disease conditions (Spearman rank’s correlation coefficient *r*_*s*_ = 0.84; *P* = 2.2 × 10^−23^; Fig. 5A). A total of 320 (73.23%) genes achieved a *P* value < 0.05 (Additional file 2: Table S13), suggesting a robust change in OS gene expression in CD.

Colocalization analysis demonstrated candidate genetic regulations of both intestinal gene expression and gut microbiota; however, little information is available on the correlative directions between the latter two. We then integrated the paired fecal metagenomic and intestinal gene expression data from the FAH-SYS cohort. Two genera and three species potentially involved in colocalized metabolic pathways were evaluated for their associations with intestinal gene expression (the “Methods” section). Five gene–microbiota pairs showed significant correlations in the FAH-SYS cohort (*P* < 0.05; Additional file 2: Table S14). Intestinal *MUC1* gene expression was upregulated in patients with CD compared with that in HCs (beta = 3.16; *P* = 6.76 × 10^−15^; Fig. 5B). *Bacillus aciditolerans*, a species from previously reported probiotics involved in myo-inositol degradation (PWY.7237), was less abundant in the CD group than in the HC group (beta =  − 2.44; *P* = 0.0026). A negative correlation between *MUC1* expression and *B. aciditolerans* abundance was observed in the HC group (beta =  − 0.093; *P* = 0.05). *E. coli* is a purine utilizer (P164.PWY) and an opportunistic pathogen associated with various immune-related diseases [67]. We identified a higher abundance of *E. coli* in CD while it was associated with a lower level of *PRKAB1* gene expression (beta =  − 0.012; *P* = 0.039 in HCs; Fig. 5C). Meanwhile, we observed an increasing trend in *S. cerevisiae* abundance in the FAH-SYS CD group (beta = 0.38; *P* = 0.11), which participates in L-isoleucine biosynthesis (PWY.5101). The association between *S. cerevisiae* and *CD40* expression was also insignificant (Fig. 5D). Notably, the lack of a significant correlation between these intestinal gene expressions and microbiota in CD was likely due to dysbiosis in the disease with disrupted host–microbiota interactions [68]. Nevertheless, the results from an external cohort were consistent with eQTL–mbQTL colocalization. This provides complementary evidence of the CD-causal effect of *MUC1* expression and the protective role of *PRKAB1* expression upon interaction with gut microbiota.

## Discussion

To the best of our knowledge, this study is the first to leverage a multi-omics integration method to detect putative causal OS genes and the underlying mechanisms in CD using blood and intestinal tissues. We identified 438 OS-related DEGs out of 817 potential genes in CD in a sizable meta-analysis of intestinal transcriptome data and successfully validated these DEGs in our cohort. Integration of GWAS with the eQTLs and mQTLs of these DEGs from the peripheral blood prioritized five putative OS genes and their regulatory elements associated with CD onset: *BAD*, *SHC1*, *STAT3*, *MUC1*, and *GPX3*. Moreover, the integration of intestinal eQTL data also identified five candidate causal genes, of which *MUC1*, *CD40*, and *PRKAB1* were involved in intestinal gene–microbiota interactions through further colocalization analysis. Differentially expressed OS genes in CD can either be a cause or a collateral effect of intestinal inflammation; our study is therefore fundamental as an attempt to fill the gaps in our understanding of discriminating between either causally or remotely involved OS genes and pinpointing the relevant interactions in CD in a genomic context.

Recently, more and more blood-based biomarkers are being used to diagnose, monitor, and predict IBD activities, and blood tissue may serve as a valuable proxy in terms of characterizing genetic effects on gene expression and understanding the complex etiology of IBD. Using an SMR analysis with blood tissue, we detected five putative causal associations between OS genes and CD susceptibility through genetically epigenomic and transcriptomic regulation, suggesting a vital role of epigenetic factors and gene expression in the disease onset. Among these genes (*BAD*, *SHC1*, *STAT3*, *MUC1*, and *GPX3*), the causal roles of *STAT3* and *MUC1* have been extensively characterized in CD [69–71]. For instance, *STAT3* plays a crucial role in many cellular processes, including cell growth and apoptosis in response to cellular stimuli, and has been regarded as a CD susceptibility gene according to previous GWASs [72–74]. Moreover, a T cell-specific *STAT3* deletion has been reported to ameliorate dextran sulfate sodium-induced colitis in mice by reducing the inflammatory response [75]. Importantly, our study confirmed that an increased transcript level of *STAT3* may lead to an increased CD risk (beta_SMR_ = 0.70). Additionally, we revealed that DNAm in enhancer regions negatively regulated *STAT3* expression, suggesting a link between DNAm, *STAT3* expression, and CD risk. More importantly, another three candidate genes lacking intensive study were identified from blood tissue that might be causal to CD: *GPX3*, *SHC1*, and *BAD*. *GPX3* is involved in the redox-sensitive KEAP1-NRF2/ARE signaling system which is considered a pivotal target in maintaining cellular homeostasis under OS, inflammatory conditions, and pro-apoptotic conditions [76]. To date, studies on the *GPX3* gene, which is a target gene of NRF2, have mainly focused on cancers, including colitis-associated carcinoma; for example, *Gpx3*-deficient mice exhibited increased tumor number and inflammation, suggesting a protective role of *GPX3* in colitis-associated carcinoma [77]. Similarly, our findings indicated a negative (protective) effect of *GPX3* expression on CD susceptibility (beta_SMR_ =  − 0.15). *SHC1* is a signaling adapter molecule that is heavily understudied in CD. This gene encodes three main isoforms, and the most extended isoform (p66Shc) is a central regulator of OS in mitochondria and cells across multiple diseases [78–80]. This work showed that four DNAm sites near the promoter regions were significantly associated with *SHC1* and CD, indicating a co-regulatory pattern involving multiple epigenetic regulatory elements [81]. BAD protein is a key participant in mitochondria-dependent apoptosis and pathophysiological processes that involve the regulation of OS [82]. Although previous CD GWAS data have identified genetic variants located nearby OS genes like *SHC1* and *BAD* [83], it remains unclear whether these genes have a causal effect on CD. Based on our SMR analysis, we hypothesize that genetic variants could regulate the expression of these genes through DNA methylation, thereby affecting CD pathogenesis.

Tissue- and cell-specific gene expression has been shown to elucidate different biological molecular mechanisms [55, 84]. CD is a gastrointestinal disease, and studying its genetic effects on OS gene expression in the intestine using intestinal eQTLs (the most pertinent tissue type) may be more meaningful than that in the blood. As the intestinal barrier directly contacts with luminal microbes and oxidized compounds from external environment factors and senses the recurrent oxidative changes, OS genes in the intestine may be associated with CD through host–microbiota interactions. Our SMR-based analysis pinpointed *MUC1*, *CD40*, *PARK7*, *PRKAB1*, and *NDUFS1* as putative causal genes in intestinal tissue, of which *MUC1* was also of interest in the blood. However, the association between *MUC1* expression and CD differed in the blood compared to that in the intestine, suggesting tissue-specific effects during the onset of CD. Moreover, we identified novel genes in this context that might contribute to CD pathogenesis, such as *PRKAB1* and *NDUFS1*. Three genes, *MUC1*, *CD40*, and *PRKAB1*, were further prioritized when considering the interactions between host genetics and microbiota. *MUC1* encodes a vital constituent of mucus and is overexpressed and hypo-glycosylated in the development of inflammation and IBD given its role in regulating intestinal barrier function upon multiple stimuli, including OS [71, 85–87]. Furthermore, *Muc1* knockout mice are resistant to dextran sulfate sodium-induced acute intestinal injury [70]. This is consistent with our findings which confirmed that high expression of *MUC1* increases the risk of developing CD. Additionally, our study colocalized the genetic regulations of *MUC1* expression and gut microbiota. Microbial creatinine degradation and myo-inositol degradation shared genetic effects with *MUC1* expression, suggesting the potential interactions between the gene and microbiota. Creatinine supplementation is identified as a potential therapeutic treatment for IBD; creatinine can be degraded to creatine by gut microbiota [88]. Creatinine clearance is associated with reduced inflammation and decreased fibrosis [89]. In addition, myo-inositol derived from dietary phytate can be converted to short-chain fatty acids through colonic bacterial phytase activity [90]. *B. aciditolerans*, one of the predicted pathway-related taxa, was negatively correlated with *MUC1* expression in the FAH-SYS cohort. This suggests that high *MUC1* expression accompanied by decreased beneficial microbial activities could confer an increased risk of CD. Our study also inferred that *PRKAB1*, which encodes the regulatory subunit of AMP-activated protein kinase that monitors cellular energy status and responds to ROS [91], was a CD-protective OS gene. We suggest that genetic regulation of *PRKAB1* expression is associated with CD onset (beta_SMR_ =  − 0.30). Moreover, *E. coli* and related purine nucleobase degradation pathways may interact with host *PRKAB1* expression. IBD-associated *E. coli* strains have been reported to facilitate IBD flares [92]. A negative correlation between *PRKAB1* expression and *E. coli* was consistently observed in the FAH-SYS cohort. Interestingly, a recent study reported the role of *PRKAB1* agonists as barrier-protective therapeutic agents in IBD [93]. However, further evidence based on genetic background (such as knockout mouse models) is needed to precisely explain the potential role of *PRKAB1* in CD.

Integrating multi-omics from multiple tissues enables researchers to dissect GWAS signals, such as the prioritization of genes and disease mechanisms. Peripheral blood tissue has a less direct and significant effect on CD than intestinal tissue. However, its significance in generating epigenomic, transcriptomic, and proteomic evidence for identifying causally involved genes and therapeutically relevant targets is well recognized [19, 94, 95]. We prioritized a list of novel genes and DNAm sites for follow-up functional studies using the largest up-to-date CD GWAS and OS-targeted approach. More importantly, this is the first study providing evidence to support a causal role of OS genes interacting with the gut microbiota in intestinal tissue. Despite the moderate associations between host genetics and gut microbiota [46], we observed common genetic regulations of intestinal gene expression and bacterial metabolic potentials. Different bacteria harboring shared genomic contents can participate in the same metabolic functions [96, 97]. However, no individual taxa were significantly colocalized with the intestinal gene expression in the current analysis. This is likely owing to the low statistical power to detect zero-inflated taxa data in mbQTL studies [44]. Nevertheless, we used an external multi-omics cohort to confirm the association between the expression of these genes and pathway-related bacterial abundance. In addition, microbiota detected from fecal and intestinal tissues showed considerable differences [98–100], which might explain the small effect sizes from the intestinal gene–fecal microbiota associations.

Some limitations of this study warrant recognition. First, the meta-analysis of intestinal DEGs included different data resources (microarray and bulk RNA-seq with varying sample sizes) which could impose heterogeneity. However, we successfully replicated over 80% of the DEGs in an independent cohort with pronounced transcriptomic alterations of the OS gene family in patients with CD compared with the controls. Second, cell type-dependent eQTLs vary with disease progression [101, 102]. The eQTLs from the bulk RNA-seq limited the identification of key molecular mechanisms at the intestinal cell level (enterocytes, immunocytes, fibrocytes) related to CD. Third, we only focused on the *cis*-regions for OS genes in the analysis, despite the possibility that *trans*-eQTL SNPs (SNP and the center of the gene > 5 Mb) may have a widespread impact on regulatory networks [41]. Fourth, we used a Bayesian colocalization method which relies on an assumption that two straits share the same single genomic variant while the case of multiple causal variants is under-explored [103]. Finally, functional experiments are still needed to validate our findings. Moreover, as multiple factors can influence the expression of OS genes, we believe that integrating other omics data at different molecular levels (such as those of proteins and metabolites) with large sample sizes may lead to novel discoveries and improve the characterization of putatively involved causal mechanisms of OS in CD.

## Conclusions

This study expands our knowledge of the potential causality of OS and the underlying biological mechanisms in CD based on a multi-omics MR approach. We demonstrated that CD onset putatively results from a number of candidate OS genes through DNAm, gene expression, and interaction with gut microbiota. Host–microbiota interactions between our newly identified causal OS genes and microbial taxa and pathways are worth studying at a functional level to gain more in-depth insights into the underlying biological mechanisms. This study advances fundamental research into the role of OS in CD and pinpoints potentially novel therapeutic targets for clinical practice.

## Supplementary Information

**Additional file 1.** Supplementary Methods.

**Additional file 2: Table S1.** Characteristics of six transcriptome datasets used in the study. **Table S2.** 817 OS-related genes with a relevance score ≥ 7 obtained from GeneCards. **Table S3.** Meta-analysis of 708 differentially expressed OS genes from six datasets. **Table S4. **Cell type-specific expression analysis (CSEA) of 438 intestinal OS DEGs. **Table S5. **Summary-based Mendelian randomization (SMR) analysis from blood gene expression to CD (FDR < 0.05). **Table S6. **SMR analysis from blood DNA methylation to CD (FDR < 0.05). **Table S7. **SMR analysis from blood DNA methylation to gene expression (FDR < 0.05). **Table S8.** Regulatory component annotation of 665 DNA methylation sites. **Table S9. **Meta-analysis between GTEx and 1000IBD intestinal cis-eQTLs (FDR < 0.05). **Table S10.** SMR analysis from intestinal gene expression to CD (FDR < 0.05). **Table S11. **Colocalization analysis between gene expression and gut microbiota (PPH4 > 0.5). **Table S12. **Demographic characteristics of FAH-SYS IBD multi-omics cohort. **Table S13.** External cohort validation: intestinal DEG results between CD and HC. **Table S14.** External cohort validation: associations between intestinal gene expression and gut microbiota.

**Additional file 3: Fig. S1.** Regulatory component annotation of 665 DNA methylation (DNAm) sites. A) DNAm sites annotated with active or inactive chromatin states were enriched in different blood cell types. B) DNAm sites annotated with histone markers were enriched in different blood cell types. Reference of active chromatin states: active transcription start site (TSS)-proximal promoter states (TssA, TssAFlnk), a transcribed state at the 5′ and 3′ ends of genes showing both promoter and enhancer signatures (TxFlnk), actively transcribed states (Tx, TxWk), enhancer states (Enh, EnhG), and a state associated with zinc finger protein genes (ZNF/Rpts). Reference of inactive chromatin states: constitutive heterochromatin (Het), bivalent regulatory states (TssBiv, BivFlnk, EnhBiv), repressed polycomb states (ReprPC, ReprPCWk), and quiescent state (Quies). Reference histone marker: H3K4me1 is enriched at active and primed enhancers; H3K4me3 is a modification that is associated with transcriptionally active/poised chromatin.

**Additional file 4: Fig. S2.** Manhattan plots of colocalization between intestinal *cis*-eQTLs and mbQTLs. Six pairs of *cis*-eQTLs and mbQTLs with PPH4 > 0.5 were plotted for illustration: *MUC1*–CARNFORCAT.PWY, *MUC1*–PWWY.7237, *CD40*–PYRIDNUCSYN.PWY, *CD40*–PWY.5101, *CD40*–PWY.0845 and *PRKAB1*–P164.PWY. All microbial pathways were annotated by MetaCyc database (https://metacyc.org/). The *x*-axis shows the chromosomal positions while the *y*-axis indicates the –log10 *P* values of SNPs.

## Data Availability

The standard tools used in this study (including SMR and MeCS) are available at https://yanglab.westlake.edu.cn/software/smr. The pipelines and scripts were stored at https://github.com/EMO-Consortium/GeneticCorrelation. Six publicly available transcriptome datasets were obtained from the GEO database (https://www.ncbi.nlm.nih.gov/geo/) [30, 32, 34, 35, 37, 39]. GWAS summary statistics for CD were downloaded from ftp://ftp.sanger.ac.uk/pub/project/humgen/summary_statistics/human/2016-11-07/. Publicly available summary statistics of blood eQTLs and mQTLs were obtained from http://www.eqtlgen.org and https://yanglab.westlake.edu.cn/software/smr/#mQTLsummarydata, respectively. Intestinal eQTL data were downloaded from https://gtexportal.org/home/datasets and https://ega-archive.org/studies/EGAS00001002702 under EGAD00001006789. mbQTL data from the DMP study can be found at https://dutchmicrobiomeproject.molgeniscloud.org. Raw metagenomic data of the FAH-SYS cohort were deposited in the NCBI public repository (Bioproject #PRJNA793776). Raw intestinal RNA-seq data are available from the corresponding authors upon reasonable request.

## Abbreviations

BH: Benjamini–Hochberg
CD: Crohn’s disease
CSEA: Cell type-specific expression analysis
CSEA-DB: Cell type-Specific Enrichment Analysis DataBase
DEG: Differentially expressed gene
DMP: Dutch Microbiome Project
DNAm: DNA methylation
eQTL: Expression quantitative trait locus
FAH-SYS: First Affiliated Hospital of Sun Yat-sen University
FDR: False discovery rate
GEO: Gene Expression Omnibus
GTEx: Genotype-Tissue Expression
GWAS: Genome-wide association study
HC: Healthy control
HEIDI: Heterogeneity in dependent instruments
IBD: Inflammatory bowel disease
IVW: Inverse variance weighting
LD: Linkage disequilibrium
mbQTL: Fecal microbial QTL
MeCS: Meta-analysis of *cis*-eQTL in correlated samples
mQTL: DNA methylation QTL
MR: Mendelian randomization
OS: Oxidative stress
RNA-seq: RNA sequencing
ROS: Reactive oxygen species
SMR: Summary data-based Mendelian randomization
SNP: Single nucleotide polymorphism
TSS: Transcription start site
