# Supplementary material for: Oxidative stress gene expression, DNA methylation, and gut microbiota interaction trigger Crohn’s disease: a multi-omics Mendelian randomization study
Source: BMC Med. 2023 May 11;21:179. doi: 10.1186/s12916-023-02878-8 (PMC10173549; doi:10.1186/s12916-023-02878-8)
Supplement: Supplementary file 1 — Additional file 1. Supplementary Methods. [file 12916_2023_2878_MOESM1_ESM.pdf]

## Supplementary Methods

*Intestinal bulk RNA-seq and microarray data*

The original gene count tables from three bulk RNA-seq datasets and three gene expression microarrays were obtained from the Gene Expression Omnibus (GEO) database (accession number: GSE75214, GSE112366, GSE179285, GSE165512, GSE137344, and GSE111889) [29–39]. All gene expression data were obtained from the intestinal biopsies of patients with CD and healthy controls. R package *edgeR* was used to normalize the gene matrix of the RNA-seq data using the trimmed mean of M-values (TMM) method followed by log transformation. Microarray data were annotated in R using the platform annotation files acquired from the GEO database and log-transformed for further analysis.

*GWAS summary statistics for CD*

CD summary statistics were downloaded from ftp://ftp.sanger.ac.uk/pub/project/humgen/summary_statistics/human/2016-11-07/. This study conducted a meta-analysis with two separate GWAS, including 12,160 IBD cases/13,145 population controls and 12,882 IBD cases/21,770 population controls, the most extensive publicly available data [40]. However, our primary focus was on CD; therefore, the summary results from 12,194 CD patients and 28,072 controls based on European populations were extracted.

*Blood eQTL data*

Blood eQTL data were obtained from eQTLGen (http://www.eqtlgen.org). The eQTLGen Consortium investigated the genetics of blood gene expression in 31,684 individuals derived from 37 datasets [41]. A total of 16,987 genes and 6,298 genes were tested for *cis*-eQTL and *trans*-eQTL, respectively. The current study focused only on *cis*-eQTL summaries of OS genes.

*mQTL data*

Blood mQTL summary data were generated from a meta-analysis of two European cohorts: the Brisbane Systems Genetics Study (BSGS, n = 614) and the Lothian Birth Cohorts (LBC, n = 1,366) [19]. The criteria of *cis*-eQTL were used to select *cis*-mQTL, which constituted SNPs within 1 Mb distance from the start and end of the gene. Data were obtained from https://yanglab.westlake.edu.cn/software/smr/#mQTLsummarydata.

*Genotype-tissue expression (GTEx) data*

The GTEx portal allows access to the data of more than 50 non-diseased human tissues subjected to eQTL analysis [42]. Only the eQTL summaries of the transverse colon, sigmoid colon, and small intestine were included in our analysis because these three tissues are more relevant to CD. The V7 eQTL data were downloaded from https://gtexportal.org/home/datasets.

*1000IBD eQTL data*

These data were generated by *cis*-eQTL analysis of 299 ileal and colonic biopsies from 171 patients with IBD [43]. Tissue location, inflammation status, medication usage, and patient characteristics were corrected for *cis*-eQTL identification. The data were obtained from https://ega-archive.org/studies/EGAS00001002702 under EGAD00001006789.

*mbQTL data*

These data were generated from the Dutch Microbiome Project (DMP) study, which included 7,738 individuals to assess host genetic effects on gut microbiota [44]. We extracted summary statistics at the species level (n = 105) and functional pathways (MetaCyc pathway number n = 205) from https://dutchmicrobiomeproject.molgeniscloud.org.

*FAH-SYS IBD multi-omics cohort data*

Forty-six treatment-naïve CD patients and 44 healthy controls were prospectively recruited from the FAH-SYS IBD multi-omics cohort during their first endoscopy visit between July 2019 and September 2021. Subjects were included only if they had not taken antibiotics or probiotics over the past three months prior to sample collection. Paired intestinal biopsies and stool specimens were collected and stored at -80 °C before processing.

Total RNA was extracted from intestinal biopsies according to the standard TRIzol protocol. The quality and quantity of the RNA was checked by agarose gel

electrophoresis and the Agilent Bioanalyzer 2100 system. The qualified RNA was used to construct the libraries. The stranded poly-A selected libraries were sequenced on an Illumina NovaSeq 6000 platform and 150 bp paired-end reads were generated to a depth of ~40 million reads per library. Data processing was performed as previously described [43].

The detailed methods of fecal genomic DNA extraction and shotgun metagenomic sequencing have been described in our previous study [45].
