# Supplementary figures and images for: Oxidative stress gene expression, DNA methylation, and gut microbiota interaction trigger Crohn’s disease: a multi-omics Mendelian randomization study
Source: BMC Med. 2023 May 11;21:179. doi: 10.1186/s12916-023-02878-8 (PMC10173549; doi:10.1186/s12916-023-02878-8)

# A Chromatin state

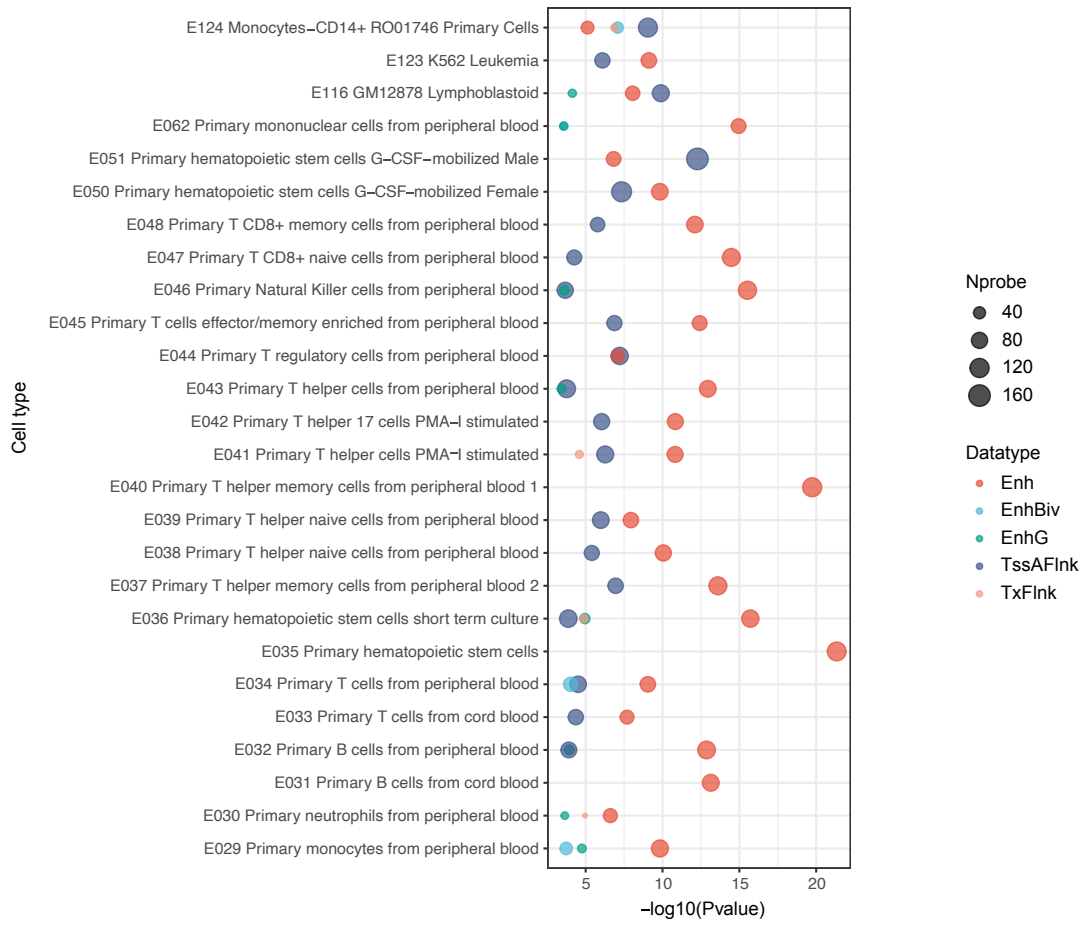

# B Histone marker

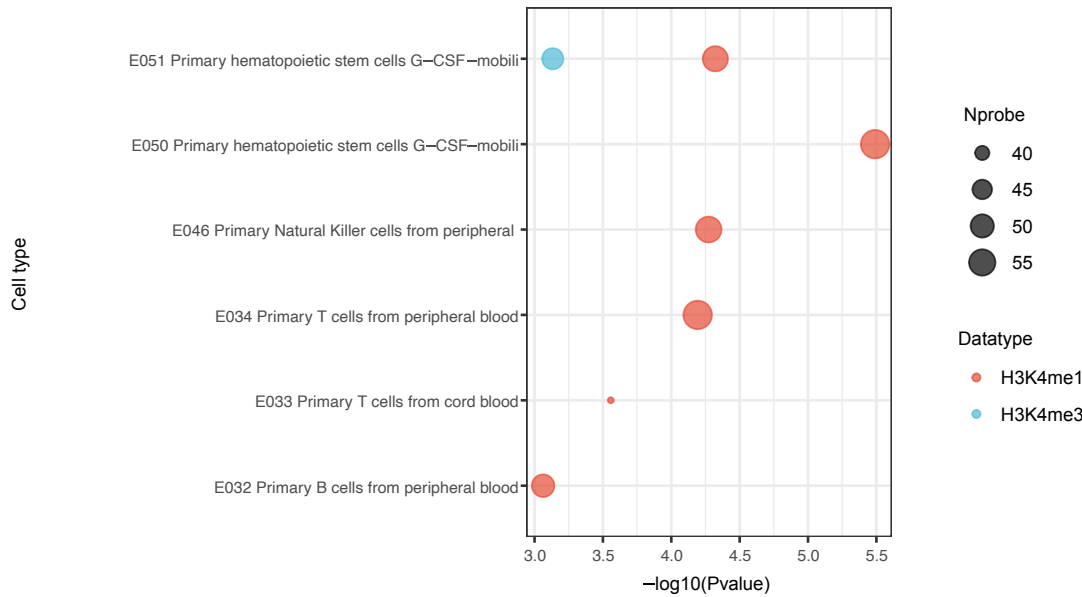

Supplement: Supplementary file 3 — Additional file 3: Fig. S1. Regulatory component annotation of 665 DNA methylation (DNAm) sites. A) DNAm sites annotated with active or inactive chromatin states were enriched in different blood cell types. B) DNAm sites annotated with histone markers were enriched in different blood cell types. Reference of active chromatin states: active transcription start site (TSS)-proximal promoter states (TssA, TssAFlnk), a transcribed state at the 5′ and 3′ ends of genes showing both promoter and enhancer signatures (TxFlnk), actively transcribed states (Tx, TxWk), enhancer states (Enh, EnhG), and a state associated with zinc finger protein genes (ZNF/Rpts). Reference of inactive chromatin states: constitutive heterochromatin (Het), bivalent regulatory states (TssBiv, BivFlnk, EnhBiv), repressed polycomb states (ReprPC, ReprPCWk), and quiescent state (Quies). Reference histone marker: H3K4me1 is enriched at active and primed enhancers; H3K4me3 is a modification that is associated with transcriptionally active/poised chromatin. [file 12916_2023_2878_MOESM3_ESM.pdf]

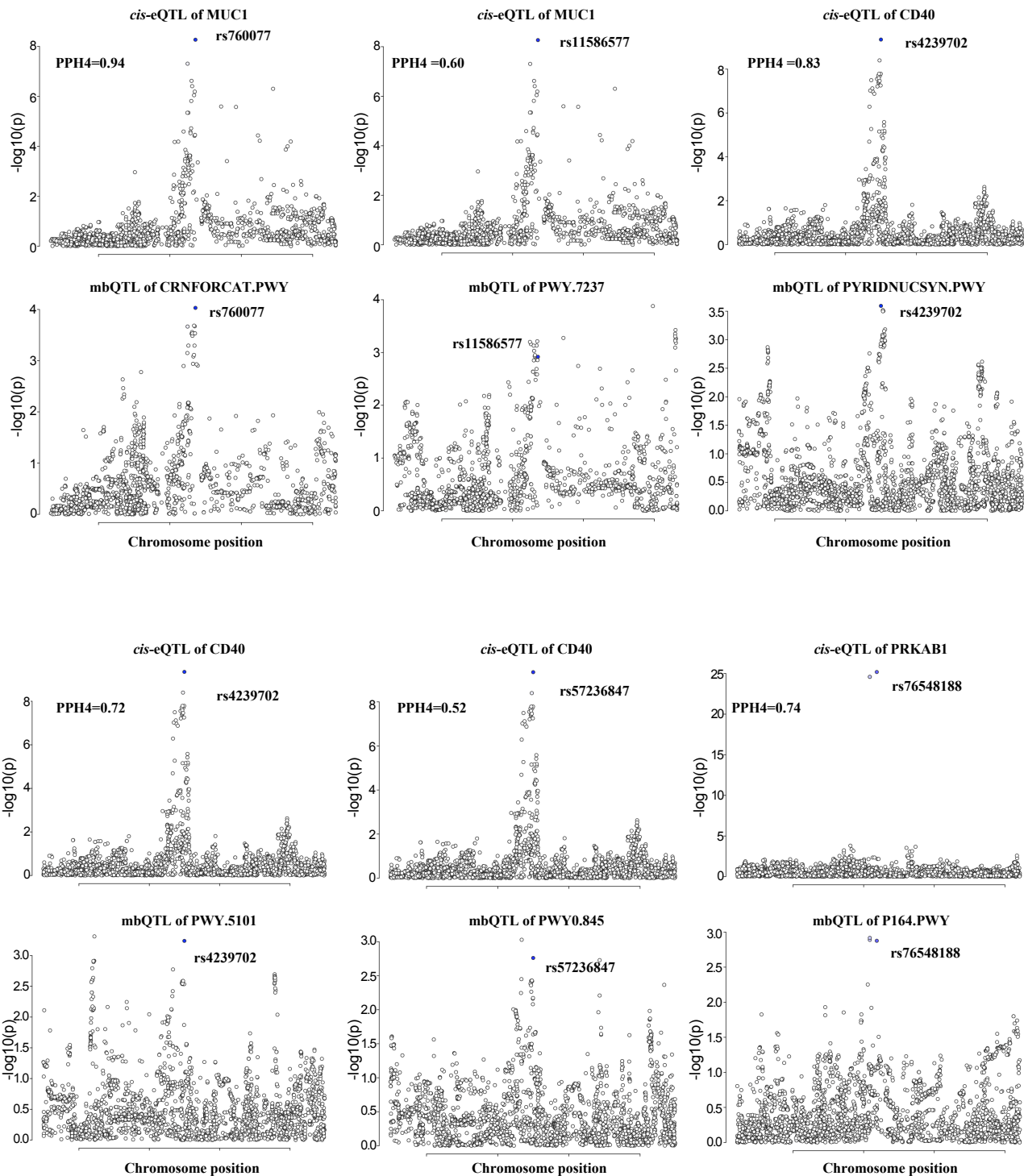

Supplement: Supplementary file 4 — Additional file 4: Fig. S2. Manhattan plots of colocalization between intestinal cis-eQTLs and mbQTLs. Six pairs of cis-eQTLs and mbQTLs with PPH4 > 0.5 were plotted for illustration: MUC1–CARNFORCAT.PWY, MUC1–PWWY.7237, CD40–PYRIDNUCSYN.PWY, CD40–PWY.5101, CD40–PWY.0845 and PRKAB1–P164.PWY. All microbial pathways were annotated by MetaCyc database (https://metacyc.org/). The x-axis shows the chromosomal positions while the y-axis indicates the –log10 P values of SNPs. [file 12916_2023_2878_MOESM4_ESM.pdf]
